# Early clinical experience with the Toumai robotic system in general surgery: a systematic review of short-term outcomes and telesurgical applications

**DOI:** 10.1007/s11701-026-03714-w

**Published:** 2026-07-27

**Authors:** Francesco Brucchi, Rita Stanco, Beatrice Sperotto, Richard Sassun, Roberto Cirocchi, Giampaolo Formisano, Paolo Pietro Bianchi, Gianlorenzo Dionigi

**Affiliations:** 1 Division of Surgery, Istituto Auxologico Italiano, IRCCS, Via Mercalli 30, Milan, Italy; 2 Department of Pathophysiology and Transplantation, University of Milan, Milan, Italy; 3 University of Pavia, Pavia, Italy; 4 General Surgery Unit, Pederzoli Hospital, Peschiera del Garda, Verona, Italy; 5 General Surgery Unit, Pio XI Hospital, Desio, Monza e Brianza, Italy; 6 Department of Medicine and Surgery, University of Perugia, Perugia, 06123 Italy; 7 Department of Surgery, Dipartimento di Scienze della Salute, ASST Santi Paolo e Carlo, University of Milan, Milan, Italy; 8 Milan, Italy

**Keywords:** General surgery, Toumai surgical robot system, Robotic-assisted surgery, Telesurgery, 5G communication

## Abstract

The Toumai Surgical Robot System is an emerging robotic platform with potential advantages related to cost and native telesurgical capability. This systematic review evaluated the early clinical experience with the Toumai system in general surgery, focusing on procedural characteristics, perioperative outcomes, and 5G remote applications. A PRISMA-compliant systematic review was conducted. MEDLINE/PubMed, EMBASE, the Cochrane Library, and Web of Science were searched through March 2026. Studies reporting general surgical procedures performed with the Toumai system and postoperative outcomes were included. Purely urological or gynaecological series, conference abstracts, narrative reviews, and preclinical studies were excluded. Risk of bias was assessed using ROBINS-I. Eight studies were included: four retrospective cohorts, three prospective clinical trials, and one case report. All were conducted in China and comprised 416 patients, of whom 329 were treated with a confirmed Toumai system; 87 patients came from a mixed-platform HPB cohort and were considered supportive, platform-non-specific evidence. Procedures included HPB surgery, gastric surgery, cholecystectomy, inguinal hernia repair, colorectal surgery, and one vascular resection. Conversions were infrequent, blood loss was low, and complications were predominantly Clavien–Dindo I–II, with no procedure-related mortality. One propensity-score-matched study found no significant short-term differences between Toumai and da Vinci Xi radical gastrectomy. Two prospective studies demonstrated 5G remote surgery for radical gastrectomy over 15 km and cholecystectomy over 70 km. On ROBINS-I assessment, most included studies were judged to be at moderate risk of bias. Early evidence suggests that the Toumai system is feasible and safe in the short term across selected general surgical procedures, with distinctive 5G telesurgical capability. Comparative effectiveness, generalisability, and cost-effectiveness remain unproven.

**Study Registration**: A review protocol was registered in PROSPERO (CRD420261428858).

**Supplementary Information:**

The online version contains supplementary material available at 10.1007/s11701-026-03714-w.

## Introduction

The use of robotic platforms for general surgery has expanded rapidly over the past two decades, reflecting the advantages reported with these technologies — magnified three-dimensional visualization, tremor filtration, and wristed instruments with multiple degrees of freedom — which together facilitate complex dissection and intracorporeal reconstruction while reducing surgical trauma [1, 2]. For more than twenty years the da Vinci Surgical System (Intuitive Surgical, approved by the FDA in 2000) has been the benchmark platform; however, its acquisition, consumable, and maintenance costs constitute a substantial barrier to wider dissemination, particularly in resource-variable settings [1, 4].

This economic pressure, together with the expiry of foundational patents, has driven the development of a new generation of robotic platforms. Among them, the Toumai Surgical Robot System (MicroPort MedBot, Shanghai, China) is the first four-arm laparoscopic surgical robot independently developed in China, having received National Medical Products Administration (NMPA) approval in February 2022 and subsequent CE marking [1, 4]. The Toumai is a master–slave system comprising a surgeon console, a patient-side cart, and a vision platform; it provides three-dimensional high-definition imaging and wristed instruments with up to 540° of articulation, seven joints and 20 degrees of freedom per arm [1, 4]. Reported distinctive features include a lower master–slave latency, a lower purchase price (approximately US$1.9 million versus US$2.8 million) and roughly half the annual maintenance cost of the da Vinci Xi, and — most notably — native support for multi-network (5G/wired/satellite) connectivity that enables remote surgery, with a bidirectional latency below 150 ms [1, 4]. The remote capability distinguishes the Toumai from most competing platforms and aligns it with the long-standing ambition of telesurgery first realised in the 2001 Lindbergh operation [3].

To date the clinical literature on the Toumai system is dominated by urology — where radical prostatectomy, partial nephrectomy, and radical cystectomy have been the most extensively studied and have already been synthesised in dedicated reviews [13, 14] — and is beginning to extend into gynaecology, including the first European robot-assisted and telerobotic total hysterectomies [17, 18]. More broadly, the robotic field is diversifying in parallel toward single-port and other emerging platforms, making the structured synthesis of early experience with each new system increasingly valuable [19]. By contrast, the experience in general surgery is comparatively recent, dispersed across heterogeneous procedures — hepato-pancreato-biliary, gastric, colorectal, biliary, abdominal-wall, and vascular — and has not been synthesised. Evaluating performance during this early implementation phase is critical for understanding the system’s safety, its procedural reach, and its potential role relative to established platforms.

This systematic review aims to consolidate the current evidence on the early experience with the Toumai Robot System in general surgery, examining procedure type, operative and console times, docking time, blood loss, conversions, postoperative complications, and length of stay, with remote (telesurgical) application analysed as a cross-cutting theme. To our knowledge, this is the first synthesis of the evidence on the Toumai system specifically in general surgery.

## Methods

### Study design

This review was conducted in accordance with the Preferred Reporting Items for Systematic Reviews and Meta-Analyses (PRISMA) statement and the recommendations of the Cochrane Handbook for Systematic Reviews of Interventions. The protocol was prospectively registered in PROSPERO (CRD420261428858). Institutional Review Board approval was not required, as the review used previously published data and did not involve human participants.

### Eligibility criteria

Studies addressing the use of the Toumai system in general surgical procedures and reporting postoperative outcomes were eligible for inclusion. Studies were excluded if they (1) did not report postoperative outcomes, (2) involved procedures outside the scope of general surgery (for example, purely urological, gynaecological, or isolated thoracic series), (3) did not report relevant outcomes, or (4) were conference abstracts, narrative reviews, editorials, or preclinical/skill-assessment studies. Given the recent and concentrated adoption of the Toumai system, potential overlap between study populations was assessed by considering authorship, institutional patient origin, and study timeframes; overlapping populations were identified and flagged to avoid duplication.

### Search strategy and extraction

**Fig. 1:**
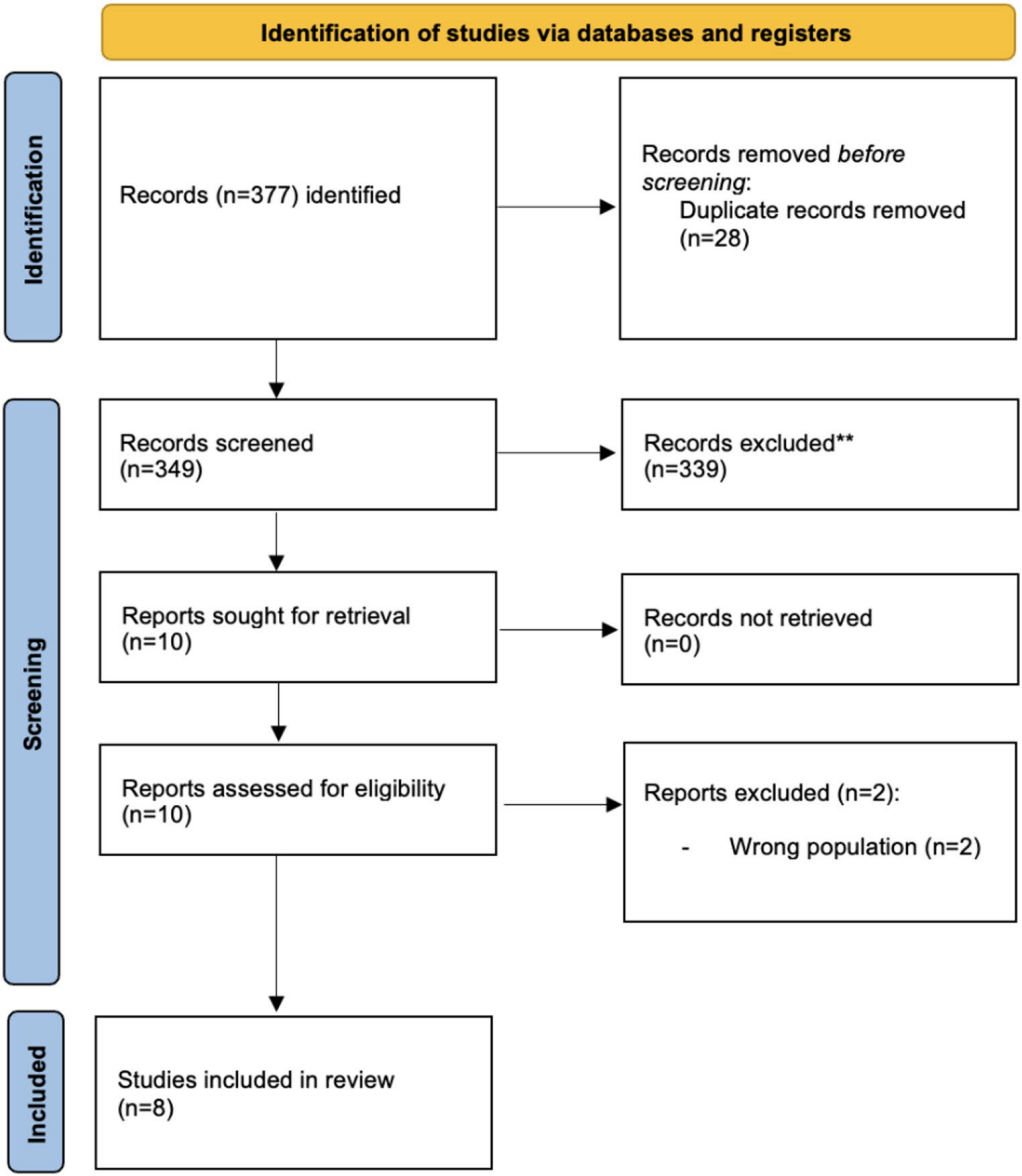
PRISMA 2020 flow diagram of study identification, screening, and inclusion

A comprehensive literature search was conducted across PubMed/MEDLINE, EMBASE, the Cochrane Library, and Web of Science from inception to March 2026, with no language restriction. Two reviewers independently screened titles and abstracts and then full texts against the eligibility criteria, and independently extracted data using a standardised, piloted extraction form; disagreements at every stage were resolved by discussion and, where necessary, adjudication by a senior author. The search combined platform and procedure terms — representatively, for PubMed: (“Toumai”[tiab] OR “MicroPort”[tiab] OR “MedBot”[tiab] OR “MT-1000”[tiab]) AND (“general surgery”[tiab] OR “gastrointestinal”[tiab] OR “hepatobiliary”[tiab] OR “pancreatic”[tiab] OR “hernia”[tiab] OR “cholecystectomy”[tiab] OR “robotic surgery”[tiab] OR “minimally invasive surgery”[tiab] OR “telesurgery”[tiab]) — with the equivalent syntax adapted to the Emtree, Cochrane, and Web of Science interfaces; the full database-specific strategies are provided in Supplementary Material. Reference lists of included articles and relevant reviews were hand-searched. The study-selection process, including the numbers of records identified, screened, assessed for eligibility, and excluded with reasons, is summarised in a PRISMA 2020 flow diagram (Fig. 1). Extracted data included country, study design, number of confirmed Toumai patients, docking time, console (cockpit) and total operative time, conversions, intraoperative blood loss, postoperative complications graded by the Clavien–Dindo classification, length of stay, and, for remote procedures, distance and network parameters (latency, jitter, packet loss).

### Quality assessment

The Cochrane Risk Of Bias In Non-randomised Studies of Interventions (ROBINS-I) tool was used to assess risk of bias in the comparative and single-arm observational studies. Case reports were not amenable to ROBINS-I evaluation and were therefore not graded. Each study was independently assessed and categorised as low, moderate, serious, or critical risk of bias, with disagreements resolved by consensus. ROBINS-I was selected because every study eligible for grading was non-randomised — comprising retrospective cohorts and prospective single-arm trials — a design mix for which the domain-based ROBINS-I framework is uniformly applicable, whereas randomised-trial instruments (e.g., RoB 2) were not; the single case report was not amenable to structured risk-of-bias grading. A formal certainty-of-evidence assessment (e.g., GRADE) was not undertaken, because the substantial clinical and methodological heterogeneity of the included studies and the consequent absence of any quantitative synthesis precluded a meaningful per-outcome rating of the overall body of evidence; the certainty of the current evidence is therefore appraised narratively.

### Statistical analysis

Findings were summarised descriptively and presented by procedure category: (I) hepato-pancreato-biliary surgery, (II) gastric surgery, (III) colorectal surgery, (IV) biliary (cholecystectomy), (V) abdominal-wall, and (VI) other procedures. Median operative and console times, blood loss, and length of stay were reported as available, together with postoperative complications. Remote (telesurgical) parameters were tabulated separately. Owing to substantial clinical and methodological heterogeneity in study designs, procedures, and reported outcomes — which precluded any valid quantitative pooling — no quantitative synthesis (meta-analysis) was performed, and a narrative synthesis was undertaken instead; comparative findings versus the da Vinci system or laparoscopy were summarised narratively.

## Results

The study-selection process is summarised in the PRISMA 2020 flow diagram (Fig. 1). Of *377* records identified and *349* screened after duplicate removal, *10* full texts were assessed for eligibility. Eight studies satisfied the inclusion criteria [5–12]: 4 retrospective cohorts (two single-centre, one multicentre series of prospectively collected data, and one propensity-score-matched comparison with the da Vinci Xi) [5, 6, 9, 11], 3 prospective single-arm or controlled clinical trials [7, 8, 10], and 1 case report [12]. All were conducted in China and together reported 416 patients, of whom 329 were treated with a confirmed Toumai system; the multicentre HPB cohort of 87 patients [6] pooled the Toumai with the Edge MP series without platform-specific reporting and is therefore treated as supportive, platform-non-specific evidence and excluded from the Toumai-specific denominator (see Sect.  3.1 and the sensitivity interpretation in the Discussion). The characteristics of the included studies are summarised in Table 1, and perioperative outcomes in Table 2. Hepato-pancreato-biliary procedures accounted for the largest number of patients (247 across both platforms; 160 confirmed Toumai), followed by gastric (103), biliary cholecystectomy (40), abdominal-wall hernia repair (16), colorectal (9), and a single vascular (inferior vena cava hemangioma) resection.

**Table 1:** Characteristics of included studies

Author, year	Center, country	Study design	Toumai patients (*n*)	Procedure category
Xu et al., 2025 [5]	PLA General Hospital, Beijing, China	Retrospective cohort	160	Hepato-pancreato-biliary
Chen et al., 2026 [6]	11 centres (multicentre), China	Retrospective cohort (prospectively collected)	87*	Hepato-pancreato-biliary
Guo et al., 2026 [7]	Fourth Hosp. Hebei Med. Univ., China	Prospective single-arm trial (FUTURE-04)	27	Gastric — 5G remote
Zhang et al., 2025 [8]	Gansu Provincial Hospital, China	Prospective single-arm exploratory	21	Gastric (12) + colorectal (9)
Tian et al., 2025 [9]	Fourth Hosp. Hebei Med. Univ., China	Retrospective cohort (PSM vs. da Vinci Xi)	64	Gastric (vs. da Vinci Xi)
Yang et al., 2025 [10]	Gansu Provincial Hospital, China	Prospective controlled trial	40†	Biliary — 5G remote cholecystectomy
Wang et al., 2026 [11]	Gansu Provincial Hospital, China	Retrospective cohort	16	Abdominal-wall (inguinal hernia, TAPP)
Sunyi et al., 2024 [12]	First Affiliated Hosp., Zhejiang Univ., China	Case report	1	Vascular (IVC hemangioma)

*Multicentre series pooling the Toumai platform with the Edge MP series; platform-specific outcomes not separately reported. †Yang et al.: 20 remote (5G) plus 20 local Toumai cholecystectomies

**Table 2:** Perioperative outcomes

Author, year	*N*	Procedure	Operative / console time (min)	Docking (min)	Conversion	Blood loss (mL)	LOS (days)
Xu 2025 [5]	160	Liver / pancreatic / biliary resections	Master–slave 114 (17–395); console: liver 109, panc. 69, biliary 150	17 (5–35)	3 (1.9%)	30 (5–300)	5 (1–27)
Chen 2026 [6]	87*	PD, distal panc., enucleation, hepatectomy, hilar CCA	PD 495; RDP 240; REN 240; hepatectomy 240; hilar CCA 420 (median)	NR	3 (3.5%)	PD 150; hepatectomy 100; hilar CCA 240	PD 15; hepatectomy 8; hilar CCA 10
Guo 2026 [7]	27	Remote radical gastrectomy (D2), 5G	Operative 193 ± 35; console 178 ± 34	15 (14–16)	0	36 ± 16	7
Zhang 2025 [8]	21	Gastrectomy (12); colorectal resection (9)	Console: gastric 121.5; colorectal 68 (median)	Gastric 17.5; colorectal 22	0	Gastric 100; colorectal 50	Gastric 9; colorectal 7
Tian 2025 [9]	64	Radical gastrectomy (vs. da Vinci Xi)	Operative 199 ± 37 (median 190)	12.4 ± 2.1	0	37 ± 22 (median 30)	8 (6–13)
Yang 2025 [10]	20 / 20	Cholecystectomy (5G remote / local)	Console 32 / 36	7.6 / 9.9	1 / 0	14 / 16	2.8 / 2.9
Wang 2026 [11]	16	Inguinal hernia repair (TAPP)	Operative 104 ± 19	NR	0	6.9 ± 3.8	3.2 ± 0.5
Sunyi 2024 [12]	1	IVC hemangioma resection	NR	NR	0	NR	5

Values are median (range) or mean ± SD as reported by the source studies. *Mixed-platform multicentre cohort (Toumai + Edge MP). CCA, cholangiocarcinoma; LOS, length of stay; NR, not reported; PD, pancreaticoduodenectomy; RDP, robotic distal pancreatectomy; REN, robotic enucleation

### Hepato-pancreato-biliary surgery

Two studies reported hepato-pancreato-biliary (HPB) procedures [5, 6]. Xu et al. retrospectively analysed 160 consecutive patients undergoing liver (*n* = 92), pancreatic (*n* = 23), and biliary (*n* = 37) procedures at a single high-volume centre, representing the first reported use of the Toumai system in HPB surgery [5]. The median positioning/docking time was 17 min (range 5–35) and the median master–slave (intra-abdominal) operating time was 114 min (range 17–395), with procedure-specific median console times of 109 min for liver, 69 min for pancreatic, and 150 min for biliary surgery. Median intraoperative blood loss was 30 mL without transfusion, and the median postoperative stay was 5 days (range 1–27). Conversion to open surgery occurred in 3 patients (1.9%), all for complex anatomy or vascular involvement rather than intraoperative emergency, and a history of prior abdominal surgery (28.8%) did not preclude completion. A single Clavien–Dindo III complication (pancreatic fistula with gastrointestinal bleeding after pancreaticoduodenectomy) was managed conservatively, with no other grade III–IV events and no in-hospital mortality; outcomes were reported as comparable to contemporaneous da Vinci procedures at the same institution, where surgeons were already proficient beyond the da Vinci learning curve [5].

As supportive but platform-non-specific evidence, Chen et al. reported a multicentre cohort of 87 patients undergoing HPB surgery with Chinese domestic platforms across 11 centres, comprising pancreaticoduodenectomy (*n* = 14), distal pancreatectomy (*n* = 9), enucleation (*n* = 11), central pancreatectomy and duodenum-preserving resection (*n* = 1 each), hepatectomy (*n* = 45), and radical resection for hilar cholangiocarcinoma (*n* = 5) [6]. Because this series pooled the Toumai platform with the Edge MP series and did not report platform-specific outcomes — and because the single completely remote pancreaticoduodenectomy (physical distance 900 km, network distance 1035 km, mean delay 21 ms, total end-to-end delay < 100 ms) was performed with the MP2000 system rather than the Toumai — these patients are not counted in the Toumai-specific denominator and the figures below should be read as describing domestic platforms collectively rather than the Toumai in isolation. Conversion to open surgery occurred in 3 patients (3.5%), all for intraoperative haemorrhage; nine patients developed clinically relevant grade B pancreatic fistula; one patient required reoperation (Clavien–Dindo ≥ III); R0 resection was achieved in all cases; and there was no 90-day mortality or readmission. Median operative times ranged from 240 min for hepatectomy to 495 min for pancreaticoduodenectomy, with median blood loss between 50 and 240 mL. The inability to isolate Toumai-specific results is carried forward as an explicit sensitivity caveat in the Discussion [6].

### Gastric surgery

Guo et al. (FUTURE-04) prospectively evaluated 5G robot-assisted remote radical gastrectomy with D2 lymphadenectomy in 27 patients (17 distal, 10 total) operated over a distance of 15 km [7]. All procedures were completed without conversion, with a 100% R0 resection rate and a mean of 38.4 retrieved lymph nodes. The mean operative time was 192.6 ± 34.8 min, the median docking time was 15 min, the mean console (robot) time was 177.7 ± 34.2 min, and the mean blood loss was 35.9 ± 15.9 mL. Postoperative complications occurred in 18.5% of patients, all Clavien–Dindo grade I–II with no grade ≥ III events, and the median stay was 7 days. The 5G network was stable, with a total delay of 226.2 ± 4.4 ms, a round-trip network delay of 31.6 ± 3.8 ms, and packet loss below 0.1% (Table 3) [7].

**Table 3:** Telesurgical (5G remote) procedures and network parameters

Author, year	Procedure	Remote cases (*n*)	Distance	Latency	Packet loss	Conversion
Guo 2026 [7]	Radical gastrectomy	27	15 km	Total 226 ± 4 ms; round-trip 32 ± 4 ms	< 0.1%	0
Chen 2026 [6]†	Pancreaticoduodenectomy	1	1035 km (network)	Mean 21 ms; total < 100 ms	0	0
Yang 2025 [10]	Cholecystectomy	20	70 km	Mean 43.4 ms; jitter 4 ms	< 1%	1

†The single completely remote pancreaticoduodenectomy in the multicentre HPB cohort was performed with the related MP2000 domestic platform rather than the Toumai system

In the gastrointestinal series of Zhang et al., 12 patients undergoing radical gastrectomy had a median docking time of 17.5 min, a median console time of 121.5 min, a median total operative time of 255.5 min, a median blood loss of 100 mL, and a median stay of 9 days, with no conversions [8].

Tian et al. provided the only head-to-head comparison with the da Vinci system, retrospectively analysing radical gastrectomy for gastric cancer at a single high-volume centre and applying 1:1 propensity-score matching to yield 64 Toumai and 64 da Vinci Xi patients [9]. There were no significant differences between platforms in total operative time (199.4 ± 37.3 vs. 185.8 ± 24.7 min; median 190 vs. 169 min, *p* = 0.92), docking time (12.4 ± 2.1 vs. 11.8 ± 1.8 min, *p* = 0.23), dissection time (126.5 ± 29.2 vs. 116.2 ± 18.7 min, *p* = 0.098), reconstruction time (60.3 ± 14.1 vs. 57.8 ± 11.2 min, *p* = 0.44), number of retrieved lymph nodes (41.2 ± 11.7 vs. 44.8 ± 13.7, *p* = 0.31), or estimated blood loss (36.7 ± 22.3 vs. 31.6 ± 20.8 mL, *p* = 0.31). Postoperative recovery variables — time to first flatus, time to liquid diet, length of stay (median 8 days in both groups), time to ambulation, drainage volume, and pain scores on postoperative days 1–3 — likewise did not differ. Short-term complications occurred in 15.6% of Toumai and 12.5% of da Vinci patients (*p* = 1.0), were all Clavien–Dindo grade I–II, and included no grade ≥ III events, no conversions, and no serious complications in either arm; in the Toumai group these comprised two gastrointestinal bleeds, four residual abdominal infections, and four respiratory tract infections, all resolving with conservative management. The initial Toumai docking time of approximately 18 min fell below 15 min in subsequent cases, and the authors attributed the apparently flat learning curve to the team’s extensive prior da Vinci experience (> 500 cases) and the close design similarity between the two systems [9].

### Colorectal surgery

Colorectal procedures were reported within the prospective single-arm gastrointestinal study of Zhang et al., in which 9 patients underwent radical colorectal resection (predominantly right hemicolectomy and sigmoid colectomy) [8]. The median docking time was 22 min, the median console time was 68 min, the median total operative time was 188 min, the median blood loss was 50 mL, and the median postoperative stay was 7 days, with no conversions and no intraoperative organ injury. Across the gastrointestinal cohort, the most frequent complications were Clavien–Dindo grade II events related to nutritional support after extensive resection (malnutrition, hypoproteinaemia), with a single grade III lymphatic leakage in a gastrectomy patient; no system failures or mortality occurred [8].

### Biliary, abdominal-wall and other procedures

**Biliary (cholecystectomy).** Yang et al. conducted a prospective controlled trial of 5G telerobotic cholecystectomy, comparing 20 patients operated remotely over 70 km with 20 contemporaneous local Toumai cholecystectomies [10]. The surgical success rate was 95% in the remote group and 100% in the local group; the single remote conversion to laparoscopy was prompted by severe inflammation and dense adhesions rather than any system or network failure. The remote group had a significantly shorter docking time (7.6 ± 2.5 vs. 9.9 ± 3.4 min, *p* = 0.02), with no significant differences in console time (32.4 ± 12.7 vs. 36.4 ± 12.0 min, *p* = 0.32), intraoperative blood loss (14.3 ± 3.5 vs. 16.2 ± 3.2 mL, *p* = 0.07), or hospitalisation (2.8 ± 0.9 vs. 2.9 ± 0.7 days, *p* = 0.84). Complications were minor and balanced (one wound infection remotely, one episode of abdominal distension locally), and there was no mortality. Network performance and the management of a brief intraoperative signal interruption are detailed in Sect.  3.5 [10].

**Abdominal-wall (inguinal hernia repair).** Wang et al. retrospectively compared Toumai robot-assisted (*n* = 16) with conventional laparoscopic (*n* = 34) transabdominal preperitoneal (TAPP) inguinal hernia repair [11]. Baseline characteristics (sex, age, weight, hernia classification) were comparable. The robotic group had a significantly longer operative time (104.2 ± 18.9 vs. 90.2 ± 22.3 min, *p* = 0.027) but significantly less intraoperative blood loss (6.9 ± 3.8 vs. 12.2 ± 9.3 mL, *p* = 0.008), a shorter postoperative stay (3.2 ± 0.5 vs. 3.7 ± 0.8 days, *p* = 0.015), lower postoperative pain scores (2.1 ± 0.9 vs. 3.0 ± 1.3, *p* = 0.005), and lower subjective mental-load (ESCAM) scores (18.1 ± 12.2 vs. 30.2 ± 11.5, *p* = 0.002). No intraoperative complications occurred in either group. Over 1–3 months of follow-up, recurrence was similar (1/16, 6.3% robotic vs. 2/34, 5.9% laparoscopic), while poor incision healing (0 vs. 3 cases) and incision pain (1 vs. 4 cases) favoured the robotic approach. The authors highlighted the absence of force (haptic) feedback as the principal current limitation of the Toumai platform in this setting [11]. These results are consistent with the broader robotic and minimally invasive abdominal-wall literature, in which robotic and endoscopic approaches to inguinal and ventral hernia repair have been associated with reduced blood loss and favourable postoperative recovery relative to conventional techniques, albeit with longer operative times and unresolved cost considerations [20–22].

**Other (vascular).** Sunyi et al. reported the first documented Toumai resection of an inferior vena cava (IVC) hemangioma — an exceptionally rare retroperitoneal vascular tumour — in a 37-year-old asymptomatic woman with an incidentally discovered ~ 3.3 × 3.0 cm mass [12]. Through a transperitoneal approach in the left lateral decubitus position, the multiple-arm platform afforded the dexterity and three-dimensional visualisation required for meticulous dissection and complete tumour resection while preserving the integrity of the IVC. The patient recovered uneventfully, was discharged on postoperative day 5, and showed no recurrence on subsequent follow-up. The report also documents the platform’s remote 5G capability, the operating team having performed ten remote urological procedures at a distance of approximately 800 km, underscoring the relevance of the Toumai telesurgical module beyond the gastrointestinal field [12].

### Telesurgery and 5G remote application

Remote operation emerged as a defining feature of the Toumai experience even in this interim set (Table 3). Guo et al. demonstrated safe 5G remote radical gastrectomy over 15 km, with stable connectivity (total delay 226 ms, round-trip 32 ms, packet loss < 0.1%), no conversions, and a minimal additional workload on the operating surgeon, supported by a standby bedside surgeon, a backup console, and predefined conversion triggers [7]. A completely remote pancreaticoduodenectomy over a network distance exceeding 1000 km (mean delay 21 ms) was reported within the multicentre HPB cohort, although performed with a related domestic platform [6]. Zhang et al. likewise noted that the Toumai integrates a remote surgical module and that their group has conducted multiple 5G remote procedures since 2022 [8].

The second prospective telesurgery dataset, from Yang et al., extended remote application to biliary surgery: 20 patients underwent 5G telerobotic cholecystectomy over a distance of 70 km, the longest clinical Toumai telesurgery distance in this review [10]. The communication environment was stable, with a mean network latency of 43.4 ms (range 29.1–51.6), jitter of 4 ms, upload and download rates of 98.3 and 213 Mbps, and packet loss below 1%. During one procedure a 3-second signal interruption automatically triggered the master–slave safety mechanism, switching the robot to standby; the patient’s vital signs remained stable and the operation was completed without complication, illustrating the layered safety architecture (standby bedside team, deputy console, predefined conversion triggers) that underpins remote operation. The single conversion in this cohort was driven by intraoperative findings rather than the network. Together with the 15 km remote gastrectomy of Guo et al. and the > 1000 km network-distance pancreaticoduodenectomy reported within the multicentre HPB cohort (performed with a related domestic platform), these data establish a two-distance prospective evidence base for Toumai-based telesurgery (Table 3) [6, 7, 10].

### Comparison with other surgical systems

Direct comparative evidence in this interim set is limited. In HPB surgery, perioperative outcomes were reported as comparable to contemporaneous da Vinci procedures, with longer operative times in the most complex resections attributable to docking and extreme-angle manoeuvrability [5, 6]. A device-level comparison reported by Zhang et al. highlighted a lower purchase price and roughly half the annual maintenance cost relative to the da Vinci Xi, with otherwise similar core specifications (four arms, seven joints and 20 degrees of freedom per arm, low master–slave latency) [8]. Device specifications not derived from comparative clinical studies were taken from manufacturer documentation, and the source of each specification is indicated in the supplementary device-comparison table.

The most robust comparative signal came from Tian et al., whose propensity-score-matched analysis of radical gastrectomy found no statistically significant difference between the Toumai and the da Vinci Xi across operative, docking, dissection, and reconstruction times, lymph-node yield, blood loss, length of stay, pain scores, or complications, with no conversions in either arm [9]. A device-level comparison from the same body of work, together with the specifications summarised by Zhang et al., positions the Toumai as broadly equivalent to the da Vinci Xi in core architecture (four arms, seven joints and 20 degrees of freedom per arm, low master–slave latency), with intelligent smoke evacuation, multi-network telesurgery support, a lower purchase price, and roughly half the annual maintenance cost [8, 9]. One point requires careful interpretation: the manufacturer and the device comparison credit the Toumai with a force-sensing capability (a force-sensing trocar that registers applied force at the instrument), whereas the hernia series of Wang et al. reports the absence of reliable, clinically usable haptic feedback at the console [9, 11]. These statements are not necessarily contradictory — a sensor that measures force technically is not the same as feedback the surgeon can perceive and act on intraoperatively, and the discrepancy may also reflect differences in software version, instrument set, or the specific configuration evaluated. The literature therefore supports, at most, a declared technical force-sensing function whose clinical translation into usable haptics remains unproven; against conventional laparoscopy, the practical shortcoming reported was reliance on visual rather than tactile cues [11].

### Quality assessment

Risk of bias was assessed independently by two reviewers with the ROBINS-I tool; the domain-level and overall judgements are presented in Table 4. Overall risk of bias was moderate for six of the seven non-randomised studies and serious for the multicentre mixed-platform cohort [6], in which the inability to classify the intervention at the platform level (Toumai versus Edge MP) drove the rating. The retrospective cohorts [5, 9, 11] were principally limited by confounding and by bias in participant selection, although propensity-score matching mitigated baseline imbalance in the comparative gastric and hernia studies [9, 11]. The prospective trials [7, 8, 10] were constrained chiefly by their single-arm or single-centre design and small samples; the telesurgery trials nonetheless incorporated prospective registration and pre-specified safety endpoints [7, 10]. The single case report [12] was not amenable to ROBINS-I grading. Overall, the studies maintained acceptable methodological standards for an early-implementation evidence base, but the predominance of small, single-centre, non-randomised studies — and the absence of blinded outcome assessment — limits the strength of any comparative inference.

**Table 4:** Risk-of-bias assessment (ROBINS-I) of the non-randomised included studies

Study	D1	D2	D3	D4	D5	D6	D7	Overall
Xu 2025 [5]	M	M	L	L	M	M	M	Moderate
Chen 2026 [6]	S	M	S	M	M	M	M	Serious
Guo 2026 [7]	M	L	L	L	L	M	L	Moderate
Zhang 2025 [8]	M	M	L	M	M	M	M	Moderate
Tian 2025 [9]	M	L	L	L	L	M	L	Moderate
Yang 2025 [10]	M	L	L	L	L	M	L	Moderate
Wang 2026 [11]	M	M	L	L	M	M	M	Moderate

L, low; M, moderate; S, serious risk of bias. Domains: D1, confounding; D2, selection of participants; D3, classification of interventions; D4, deviations from intended interventions; D5, missing data; D6, measurement of outcomes; D7, selection of the reported result. The case report [12] was not amenable to ROBINS-I grading and is not listed. Judgements reflect the review team’s independent assessment

## Discussion

This synthesis indicates that the Toumai system is feasible and, on short-term measures, apparently safe across a broadening range of general surgical procedures — hepato-pancreato-biliary, gastric, colorectal, biliary, abdominal-wall, and vascular. The available data — 329 unambiguously Toumai-treated patients within a total of 416 across eight studies — show no procedure-related mortality, infrequent conversions, low blood loss, and complications that were predominantly Clavien–Dindo grade I–II. These are encouraging early-experience signals rather than evidence of comparative effectiveness: they describe feasibility and short-term safety, not durable, generalisable, or oncologically validated equivalence. The early general-surgery experience remains weighted toward hepato-pancreato-biliary and gastric procedures, reflecting the priorities of the high-volume Chinese centres that pioneered its adoption, and extends to a single propensity-score-matched comparison with the da Vinci Xi and to two independent prospective demonstrations of 5G telesurgery.

Two features distinguish the Toumai experience. The first is its prospectively validated capacity for 5G telesurgery, now demonstrated at two clinical distances by two independent prospective trials: remote radical gastrectomy over 15 km (total delay 226 ms, packet loss < 0.1%) [7] and remote cholecystectomy over 70 km (mean latency 43.4 ms, packet loss < 1%) [10], with remote procedures over far greater network distances reported within related cohorts [6]. This capability — absent from most competing platforms — positions the Toumai not merely as a lower-cost alternative but as a potential instrument for redistributing surgical expertise toward under-served regions, contingent on robust safety protocols (standby bedside teams, deputy consoles, predefined conversion triggers, and automatic standby on signal loss, as exercised during a brief intraoperative interruption without patient harm) and on resolution of the attendant legal and regulatory questions. The second feature is economic, and here caution is essential: a lower list and maintenance price than the da Vinci Xi gives the Toumai a potential economic appeal in cost-sensitive settings, but this is not the same as a demonstrated economic advantage. No included study performed a formal cost-effectiveness analysis, and a meaningful one would have to account for annual case volume, consumable and maintenance costs, operating-room time, the cost of complications, training and depreciation, and reimbursement — none of which are available here. Acquisition price is therefore only a starting point, and several early series were moreover conducted with the platform provided at no charge, a circumstance that can both lower reported costs and introduce sponsorship-related bias [6, 8].

Two considerations temper the telesurgical findings. First, the current evidence derives entirely from highly specialised, high-volume expert centres operating under controlled conditions, with dedicated bedside and biomedical-engineering teams, backup consoles, pre-defined conversion triggers, and advanced, purpose-provisioned network infrastructure; these are not the circumstances of routine practice, and the reported performance cannot be assumed to transfer to less-resourced or non-specialised settings. Second, beyond these technical prerequisites, wider adoption of telesurgery faces substantial and largely unresolved regulatory, legal, and organisational barriers. These include the absence of harmonised regulatory approval pathways for remote operation across jurisdictions; cross-border or cross-state licensing and credentialing of the operating surgeon; the medicolegal allocation of liability when surgeon, patient, and equipment are in different locations and an adverse event or network failure occurs; informed-consent requirements specific to remote surgery; and data-protection and cybersecurity obligations, including safeguards against interception of, latency manipulation of, or malicious interference with the control link, together with guaranteed, redundant network quality-of-service and defined fail-safe behaviour. A robust framework addressing these issues, alongside independent verification of the layered safety architecture reported here, will be a prerequisite for any transition from proof-of-concept demonstrations toward routine clinical telesurgery.

The principal difference from established systems was a longer operative workflow in some series, attributable to docking and instrument exchange and likely to diminish with experience; comparative reports suggested little additional learning for surgeons already proficient with the da Vinci system, a flat learning curve corroborated by the matched gastrectomy comparison of Tian et al. [5, 8, 9]. Reported limitations of the platform include occasional instrument tremor, arm collisions in extreme working angles, and constraints at extreme arm abduction, consistent with an early-generation device undergoing iterative refinement [5, 8].

Several limitations of the evidence base must be acknowledged, and they are better read as a potential source of systematic, directional bias than as a list of isolated caveats. All included studies were conducted in China, and almost all originated from high-volume, early-adopter centres whose teams were already proficient well beyond the da Vinci learning curve; several series were run with the platform supplied at no charge. Taken together, geographical concentration, early-adopter case selection, advanced baseline robotic expertise, and possible industry support plausibly bias the available results toward favourable outcomes and limit generalisability to routine, non-specialised, or resource-variable environments and to institutions with less robotic experience. The first Toumai series outside China have since emerged, including a 132-patient radical prostatectomy experience in Morocco [15] and the first European gynaecological applications [17, 18] — which lie outside the general-surgery scope of this review but signal a broadening geographical footprint — yet none yet provides an independent, non-Chinese general-surgery comparison. Sample sizes were small and the designs predominantly retrospective or single-arm, introducing selection and confounding bias, lacking blinded outcome assessment, and precluding meta-analysis. One of the two largest cohorts pooled the Toumai with another domestic platform without platform-specific outcomes, a potential source of classification bias [6], ; as a sensitivity consideration, excluding those 87 patients leaves 329 unambiguously Toumai-treated patients, and the qualitative conclusions of this review are unchanged whether or not that cohort is counted. Population overlap was assessed across the contributing institutions: the gastric experiences from the Fourth Hospital of Hebei Medical University (Tian, matched gastrectomy; Guo, remote gastrectomy) and from Gansu Provincial Hospital (Zhang, mixed gastrointestinal; Yang, cholecystectomy; Wang, hernia) address distinct procedures and time windows with no evident patient duplication, although shared institutional origin warrants caution. Follow-up was short and cost-effectiveness data were absent, and the absence of randomised comparisons restricts conclusions about relative effectiveness.

Taken together, the now-complete study set sharpens three conclusions. First, comparative effectiveness: the matched gastrectomy series of Tian et al. provides the best available — but still single, retrospective — signal that, in experienced hands, short-term Toumai outcomes do not differ significantly from those of the da Vinci Xi, while the hernia comparison of Wang et al. shows advantages over conventional laparoscopy in blood loss, pain, mental load, and length of stay, offset by a longer operative time. Second, telesurgery: the evidence base is no longer a single demonstration but a two-distance prospective dataset (15 km gastrectomy and 70 km cholecystectomy), reinforced by reports of far greater network distances and by prospective 5G remote radical prostatectomy in urology [16], strengthening the case for the Toumai as a telesurgical platform. Third, haptics: the role of force feedback remains unsettled, with one comparative device analysis crediting the Toumai with force-sensing capability while the hernia series identifies the absence of reliable haptic feedback as a limitation requiring compensation through visual cues and surgeon experience [9, 11]; this discrepancy should be resolved by independent, procedure-specific evaluation.

## Conclusion

This synthesis of eight studies and 416 patients (329 unambiguously Toumai-treated) suggests that the Toumai Surgical Robot System appears feasible and safe in the short term across hepato-pancreato-biliary, gastric, colorectal, biliary, abdominal-wall, and vascular surgery, with consistent short-term safety signals, infrequent conversions, low blood loss, and predominantly low-grade complications. A single propensity-score-matched comparison found no significant short-term difference from the da Vinci Xi in radical gastrectomy, and against laparoscopy the system reduced blood loss, pain, and length of stay in inguinal hernia repair at the cost of longer operative time; these findings are encouraging but do not establish comparative effectiveness. They should be read as reflecting technical feasibility and short-term safety rather than proven clinical efficacy or equivalence to established robotic systems. The system is further distinguished by a prospectively validated, two-distance capacity for 5G telesurgery and by a potential — not yet demonstrated — economic appeal. The evidence remains geographically confined to China, drawn largely from high-volume early-adopter centres, and methodologically limited by small, mostly non-randomised, short-term studies. Prospective, ideally randomised, multicentre trials with longer follow-up and formal cost-effectiveness analyses are needed before the Toumai can be considered comparable to established platforms in robotic general surgery. Priorities for future research include multicentre and international validation beyond high-volume Chinese early-adopter centres, randomised or rigorously matched comparative studies against established robotic and laparoscopic approaches, standardised reporting of learning curves, longer-term oncological and functional outcomes, and independent, formal cost-effectiveness analyses; the regulatory, licensing, medicolegal, and cybersecurity questions raised by telesurgery must be resolved in parallel.

## Supplementary Information

Below is the link to the electronic supplementary material.

Supplementary Material 1

## Data Availability

No datasets were generated or analysed during the current study.

## Declarations

### AI tools

AI-assisted tools were used only for lexical and stylistic polishing. All scientific content, data interpretation, and final decisions were performed and verified by the authors, who take full responsibility for the manuscript.

## References

[1] Sheetz KH, Claflin J, Dimick JB (2020) Trends in the adoption of robotic surgery for common surgical procedures. JAMA Netw Open 3:e1918911 31922557 10.1001/jamanetworkopen.2019.18911 PMC6991252

[2] Hays SB, Rojas AE, Hogg ME (2024) Robotic pancreas surgery for pancreatic cancer. Int J Surg 110:6100–6110 37988409 10.1097/JS9.0000000000000906 PMC11486949

[3] Marescaux J, Leroy J, Gagner M et al (2001) Transatlantic robot-assisted telesurgery. Nature 413:379–380 11574874 10.1038/35096636

[4] Page MJ, McKenzie JE, Bossuyt PM et al (2021) The PRISMA 2020 statement: an updated guideline for reporting systematic reviews. BMJ 372:n71 33782057 10.1136/bmj.n71 PMC8005924

[5] Xu Y, Zhao Z, Zhang X et al (2025) Robotic hepatopancreatobiliary surgery using the Toumai^®^ system: initial experience and technical considerations from a single center. Updates Surg 77:2183–2189 41082093 10.1007/s13304-025-02404-w

[6] Chen WB, Zou WB, Zhang M et al (2026) Robotic hepatobiliary and pancreatic surgery using the Chinese domestic robotic platforms: a multicenter retrospective cohort study on prospectively collected data. Int J Surg 112:7425–7434 10.1097/JS9.0000000000004106 41295900

[7] Guo H, Tian Y, Ding P et al (2026) Safety and feasibility of robot-assisted remote radical gastrectomy for gastric cancer based on 5G communication technology (FUTURE-04): a prospective, single-arm clinical trial. Gastric Cancer 29:238–249 41251879 10.1007/s10120-025-01687-7

[8] Zhang Z, Zhan W, Tian H et al (2025) An initial exploratory clinical study and outcome assessment of gastrointestinal surgeries using advanced robotic-assisted techniques. Surg Endosc 39:766–775 39572427 10.1007/s00464-024-11398-2

[9] Tian Y, Yang J, Guo H et al (2025) Safety and efficacy of the Toumai robotic assisted versus the DaVinci robotic assisted radical gastrectomy for patients with gastric cancer. BMC Surg 25:544 41239366 10.1186/s12893-025-03273-1 PMC12619376

[10] Yang J, Zhan W, Zhang Z et al (2025) The safety and feasibility of telerobotic cholecystectomy via a 5G network: a prospective controlled clinical trial. Surg Endosc 39:7336–7346 40877633 10.1007/s00464-025-12005-8

[11] Wang Y, Guo C, Zhang M et al (2026) Comparative outcomes of Toumai robotic and laparoscopic transabdominal preperitoneal inguinal hernia repair in a retrospective cohort. Sci Rep 16:9124 41691012 10.1038/s41598-026-39829-1 PMC12996340

[12] Sunyi Y, Yi Z, He A et al (2024) Inferior vena cava hemangioma resected using a novel Toumai robotic surgical platform. J Vasc Surg Cases Innov Tech 10:101403 38435787 10.1016/j.jvscit.2023.101403 PMC10907154

[13] Zhang C, Wang J (2025) Evaluating the Toumai MT-1000 for urologic surgery: a systematic review and single-arm meta-analysis with remote and on-site experiences. J Robot Surg 20(1):5 41217680 10.1007/s11701-025-02968-0

[14] Tan C, Wang B, Cui W et al (2024) Robotic urologic surgery using the Toumai MT-1000 Endoscopic Surgical System: a single-center prospective analysis. Transl Androl Urol 13(12):2748–2756 39816233 10.21037/tau-24-451 PMC11732303

[15] Et-Touzani R, Aoid I, Zemrag J et al (2026) Robotic-assisted radical prostatectomy using the Toumai system: initial single-center experience in Morocco. J Robot Surg 20(1):567 42223781 10.1007/s11701-026-03529-9

[16] Ye S, Peng D, Zhu L et al (2025) 5G-remote radical prostatectomy under novel robotic systems: a prospective comparative cohort study with local surgeries. Prostate Cancer Prostatic Dis. 10.1038/s41391-025-01004-4 40721878 10.1038/s41391-025-01004-4

[17] Pasquini P, Pazzaglia E, Jamaer E et al (2026) A pilot investigation of robot-assisted total hysterectomy using the Toumai Laparoscopic Surgical Robot System. Minim Invasive Ther Allied Technol 35(3):203–209 41820202 10.1080/13645706.2026.2643794

[18] Pazzaglia E, Pasquini P, Jamaer E et al (2025) Pioneering telesurgery in gynecology: the first European case of total hysterectomy. J Robot Surg 19(1):460 40779084 10.1007/s11701-025-02638-1 PMC12334479

[19] Brucchi F, Montroni I, Cirocchi R et al (2025) A systematic review of the Da Vinci Single-Port system (DVSP) in the context of colorectal surgery. Int J Colorectal Dis 40(1):83 40175572 10.1007/s00384-025-04878-x PMC11965226

[20] Brucchi F, Boni L, Cassinotti E et al (2025) Short-term outcomes of minimally invasive endoscopic onlay repair for diastasis recti and ventral hernia repair: a systematic review and meta-analysis. Surg Endosc 39(3):1490–1500 39920372 10.1007/s00464-025-11555-1 PMC11870909

[21] Brucchi F, Lauricella S, Esposito S et al (2025) Optimizing robotic approach to ventral hernia repair: an updated systematic review and meta-analysis between preperitoneal versus retromuscular repair. J Robot Surg 20(1):101 41428244 10.1007/s11701-025-03076-9 PMC12722465

[22] Brucchi F, De Troyer A, Sassun R, Dionigi G, Muysoms F (2025) Comparison of robot-assisted enhanced-view totally extraperitoneal (eTEP) and transabdominal retromuscular (TARM/TARUP) ventral hernia mesh repair: a systematic review and meta-analysis. J Abdom Wall Surg 4:14723 40689023 10.3389/jaws.2025.14723 PMC12270933

