# Supplementary material for: Early clinical experience with the Toumai robotic system in general surgery: a systematic review of short-term outcomes and telesurgical applications
Source: J Robot Surg. 2026 Jul 27;20(1):765. doi: 10.1007/s11701-026-03714-w (PMC13402254; doi:10.1007/s11701-026-03714-w)
Supplement: Supplementary file 1 — Supplementary Material 1 [file 11701_2026_3714_MOESM1_ESM.docx]

**Supplementary Material**

*Early Clinical Experience with the Toumai Robotic System in General Surgery: A Systematic Review of Short-Term Outcomes and Telesurgical Applications*

This document contains the supplementary methods, tables, and figure referenced in the main manuscript. Items shown in red are placeholders to be finalised by the authors (e.g., PROSPERO ID, PRISMA record counts, list of full-text exclusions).

Supplementary Methods

## S1. Protocol and registration

This review was conducted in accordance with the PRISMA 2020 statement and the Cochrane Handbook for Systematic Reviews of Interventions. The protocol was prospectively registered in PROSPERO. No ethical approval was required, as only previously published, aggregate data were used. Any deviations from the protocol are reported in the main manuscript.

## S2. Eligibility criteria (PICOS)

Studies were selected against the PICOS framework summarised in Table S2.

**Table S2 |** PICOS eligibility criteria.

| **Parameter** | **Inclusion criteria** | **Exclusion criteria** |
| --- | --- | --- |
| **Population** | Patients of any age undergoing a general-surgery procedure (hepato-pancreato-biliary, gastric, colorectal, biliary, abdominal-wall, or other intra-abdominal/vascular procedure within the general-surgery remit). | Purely urological, gynaecological, thoracic, or otolaryngological series; non-human/preclinical studies. |
| **Intervention** | Procedure performed with the Toumai (MicroPort MedBot, MT-1000) robotic surgical system, including locally controlled and 5G/wired telerobotic procedures. | Procedures performed exclusively with other robotic platforms; studies in which Toumai-specific data cannot be identified are eligible only as supportive evidence (see main text). |
| **Comparator** | Any or none (single-arm series accepted); where present, da Vinci or conventional laparoscopy. | — |
| **Outcomes** | At least one reported perioperative or postoperative outcome (operative/console/docking time, conversion, blood loss, complications, length of stay, and — for remote cases — network parameters). | Studies reporting no postoperative outcome. |
| **Study design** | Randomised trials, prospective or retrospective cohorts, case series, and case reports. | Conference abstracts, narrative reviews, editorials, protocols, and preclinical/skill-assessment studies. |

## S3. Full electronic search strategies

Databases were searched from inception to March 2026 with no language or date restriction. The exact strings run in each interface are reproduced below; results were exported to a reference manager and de-duplicated before screening.

**PubMed / MEDLINE**

("Toumai"[tiab] OR "MicroPort"[tiab] OR "MedBot"[tiab] OR "MT-1000"[tiab] OR "MT1000"[tiab]) AND ("general surgery"[tiab] OR "gastrointestinal"[tiab] OR gastrectomy[tiab] OR "hepatobiliary"[tiab] OR hepatectomy[tiab] OR pancreat*[tiab] OR cholecystectomy[tiab] OR hernia[tiab] OR colorectal[tiab] OR colectomy[tiab] OR "robotic surgery"[tiab] OR "robot-assisted"[tiab] OR "minimally invasive surgery"[tiab] OR telesurgery[tiab] OR "remote surgery"[tiab])

**EMBASE (Elsevier, Emtree + free text)**

(toumai:ab,ti OR microport:ab,ti OR medbot:ab,ti OR 'mt 1000':ab,ti OR mt1000:ab,ti) AND ('general surgery'/exp OR 'gastrointestinal surgery'/exp OR gastrectomy:ab,ti OR 'hepatobiliary':ab,ti OR hepatectomy:ab,ti OR pancreat*:ab,ti OR cholecystectomy:ab,ti OR hernia:ab,ti OR colorectal:ab,ti OR colectomy:ab,ti OR 'robotic surgery'/exp OR 'robot assisted surgery'/exp OR 'minimally invasive surgery'/exp OR telesurgery:ab,ti OR 'remote surgery':ab,ti)

**Cochrane Library (CENTRAL)**

(Toumai OR MicroPort OR MedBot OR "MT-1000" OR MT1000):ti,ab,kw AND ("general surgery" OR gastrointestinal OR gastrectomy OR hepatobiliary OR hepatectomy OR pancreat* OR cholecystectomy OR hernia OR colorectal OR colectomy OR "robotic surgery" OR "robot-assisted" OR "minimally invasive surgery" OR telesurgery OR "remote surgery"):ti,ab,kw

**Web of Science (Core Collection)**

TS=((Toumai OR MicroPort OR MedBot OR "MT-1000" OR MT1000) AND ("general surgery" OR gastrointestinal OR gastrectomy OR hepatobiliary OR hepatectomy OR pancreat* OR cholecystectomy OR hernia OR colorectal OR colectomy OR "robotic surgery" OR "robot-assisted" OR "minimally invasive surgery" OR telesurgery OR "remote surgery"))

## S4. Selection process

Two reviewers independently screened titles/abstracts and then full texts against the eligibility criteria. Disagreements were resolved by discussion and, where necessary, by a senior author. Potential population overlap between studies from the same institutions was examined by comparing authorship, recruiting centre, procedure type, and enrolment window; overlapping reports were excluded to avoid double-counting (none required exclusion among the included set, although the shared institutional origin of several gastric series is noted in the main Discussion).

## S5. Data items

Data were extracted independently by two reviewers using the standardised form in Table S5-form. Where studies reported both mean ± SD and median (range), both were captured; the value used in the synthesis tables is indicated in the table footnotes of the main manuscript.

**Table S5-form |** Standardised data-extraction template.

| **Field** | **Description** |
| --- | --- |
| Study identifiers | First author, year, journal, country, recruiting centre(s), enrolment period. |
| Design | Study type; prospective/retrospective; comparative/single-arm; registration. |
| Platform | Confirmed Toumai vs mixed/other domestic platform; instrument set; software where reported. |
| Population | Number of Toumai patients; procedure type(s); indication; key baseline characteristics. |
| Operative metrics | Docking time; console (cockpit) time; total operative time; intraoperative blood loss. |
| Safety | Conversions; intraoperative complications; postoperative complications (Clavien–Dindo); reoperation; mortality; readmission. |
| Recovery | Length of stay; time to flatus/diet/ambulation; pain scores. |
| Telesurgery | Physical and network distance; latency; jitter; up/download rate; packet loss; safety events and handling. |
| Comparator data | Corresponding values for da Vinci or laparoscopy where reported, with p-values. |

## S6. Risk-of-bias assessment

Two reviewers independently applied the ROBINS-I tool to each non-randomised comparative or single-arm study across seven domains (D1 confounding; D2 selection of participants; D3 classification of interventions; D4 deviations from intended interventions; D5 missing data; D6 measurement of outcomes; D7 selection of the reported result), with an overall judgement of low, moderate, serious, or critical risk. ROBINS-I was chosen because every study eligible for grading was non-randomised (retrospective cohorts and prospective single-arm trials), for which the domain-based ROBINS-I framework is uniformly applicable, whereas a randomised-trials instrument (e.g., RoB 2) was not applicable. The case report was not amenable to ROBINS-I grading. Domain-level judgements and the rationale supporting each overall rating are given in Table S6; the summary appears as Table 4 in the main manuscript. No formal certainty-of-evidence (GRADE) assessment was performed, given the substantial clinical and methodological heterogeneity and absence of pooled effect estimates; this is acknowledged as a limitation.

Table S1. Detailed characteristics of included studies

| **Study** | **Centre, country** | **Period** | **Design** | **Platform** | **Procedures (n)** | **Toumai n** | **Comparator** |
| --- | --- | --- | --- | --- | --- | --- | --- |
| Xu et al., 2025 [5] | Gansu Provincial Hospital, China | NR | Retrospective cohort, single-centre | Toumai | Liver 92, pancreatic 23, biliary 37 | 160 | da Vinci (narrative) |
| Chen et al., 2026 [6] | 11 centres, China | NR | Retrospective, multicentre (prospectively collected) | Toumai + Edge MP* | PD 14, distal panc. 9, enucleation 11, central panc. 1, duodenum-preserving 1, hepatectomy 45, hilar CCA 5 | 87* | None (mixed) |
| Guo et al., 2026 [7] | Fourth Hosp. Hebei Med. Univ., China | NR | Prospective single-arm trial (FUTURE-04) | Toumai | Radical gastrectomy (17 distal, 10 total), D2 | 27 | None (remote) |
| Zhang et al., 2025 [8] | Gansu Provincial Hospital, China | NR | Prospective single-arm, exploratory | Toumai | Gastrectomy 12; colorectal resection 9 | 21 | None |
| Tian et al., 2025 [9] | Fourth Hosp. Hebei Med. Univ., China | Jan 2022–Mar 2024 | Retrospective cohort, propensity-matched | Toumai | Radical gastrectomy | 64 | da Vinci Xi (n=64) |
| Yang et al., 2025 [10] | Gansu Provincial Hospital (+ New District Branch), China | May–Oct 2023 | Prospective controlled trial | Toumai | Cholecystectomy (remote 70 km / local) | 40 | Local Toumai (n=20) |
| Wang et al., 2026 [11] | Gansu Provincial Hospital, China | Jan 2022–Oct 2023 | Retrospective cohort | Toumai | Inguinal hernia repair (TAPP) | 16 | Laparoscopy (n=34) |
| Sunyi et al., 2024 [12] | First Affiliated Hosp., Zhejiang Univ., China | NR | Case report | Toumai | IVC hemangioma resection | 1 | None |

*PD, pancreaticoduodenectomy; CCA, cholangiocarcinoma; IVC, inferior vena cava; NR, not reported. *Mixed-platform cohort (Toumai + Edge MP); not counted in the Toumai-specific denominator.*

Table S2. Full comparative statistics (Toumai vs comparator)

| **Variable** | **Group A** | **Group B** | **p** |
| --- | --- | --- | --- |
| **Tian et al. [9] — Toumai (n=64) vs da Vinci Xi (n=64); mean ± SD** | | | |
| Total operative time (min) | 199.4 ± 37.3 (median 190) | 185.8 ± 24.7 (median 169) | 0.92 |
| Docking time (min) | 12.4 ± 2.1 | 11.8 ± 1.8 | 0.231 |
| Dissection time (min) | 126.5 ± 29.2 | 116.2 ± 18.7 | 0.098 |
| Reconstruction time (min) | 60.3 ± 14.1 | 57.8 ± 11.2 | 0.439 |
| Retrieved lymph nodes (n) | 41.2 ± 11.7 | 44.8 ± 13.7 | 0.313 |
| Estimated blood loss (mL) | 36.7 ± 22.3 (median 30) | 31.6 ± 20.8 (median 20) | 0.313 |
| Length of stay (days), median | 8 (6–13) | 8 (6–12) | 0.690 |
| Overall complications, n (%) | 10 (15.6) | 8 (12.5) | 1.00 |
| **Wang et al. [11] — Toumai robot (n=16) vs laparoscopy (n=34); mean ± SD** | | | |
| Operative time (min) | 104.2 ± 18.9 | 90.2 ± 22.3 | 0.027 |
| Intraoperative blood loss (mL) | 6.9 ± 3.8 | 12.2 ± 9.3 | 0.008 |
| Length of stay (days) | 3.2 ± 0.5 | 3.7 ± 0.8 | 0.015 |
| Postoperative pain score | 2.1 ± 0.9 | 3.0 ± 1.3 | 0.005 |
| Subjective mental load (ESCAM) | 18.1 ± 12.2 | 30.2 ± 11.5 | 0.002 |
| **Yang et al. [10] — 5G remote (n=20) vs local Toumai cholecystectomy (n=20)** | | | |
| Surgical success (%) | 95 | 100 | NR |
| Docking time (min), mean ± SD | 7.6 ± 2.5 | 9.9 ± 3.4 | 0.02 |
| Console time (min), mean ± SD | 32.4 ± 12.7 | 36.4 ± 12.0 | 0.32 |
| Intraoperative blood loss (mL) | 14.3 ± 3.5 | 16.2 ± 3.2 | 0.07 |
| Hospitalisation (days) | 2.8 ± 0.9 | 2.9 ± 0.7 | 0.84 |
| Conversion (n) | 1 | 0 | 1.00 |

*Group A/B as defined in each shaded section header. NR, not reported.*

# Table S3. Postoperative complications by study and Clavien–Dindo grade

| **Study** | **n with complications** | **Clavien–Dindo distribution** | **Specific events** |
| --- | --- | --- | --- |
| Xu et al. [5] | 1 (0.6%) | 1 × grade III; no IV; no mortality | Pancreatic fistula with gastrointestinal bleeding after PD; managed conservatively |
| Chen et al. [6]* | See note | 1 reoperation (≥III); 9 grade B POPF | Mixed-platform cohort; 9 clinically relevant pancreatic fistulas (grade B); 1 reoperation; no 90-day mortality or readmission; R0 in all |
| Guo et al. [7] | 5 (18.5%) | All grade I–II | Remote gastrectomy cohort; individual events not specified |
| Zhang et al. [8] | See note | Predominantly II; 1 × III | Grade II nutritional-support events (malnutrition, hypoproteinaemia); 1 grade III lymphatic leakage; no conversions |
| Tian et al. [9] | 10 (15.6%) | All grade I–II; 0 ≥III | 2 gastrointestinal bleeds, 4 residual abdominal infections, 4 respiratory tract infections (all conservative). da Vinci arm: 8 (12.5%) — 4 bleeds, 1 DVT, 2 respiratory |
| Yang et al. [10] | 1 remote / 1 local | Grade I–II | 1 wound infection (remote); 1 abdominal distension (local); both resolved |
| Wang et al. [11] | 0 intraoperative | — | No intraoperative complications; at follow-up: recurrence 1 (6.3%), poor incision healing 0, incision pain 1 (vs laparoscopy 2, 3, 4 respectively) |
| Sunyi et al. [12] | 0 | — | Uneventful recovery; discharged on POD 5; no recurrence |

*POPF, postoperative pancreatic fistula; DVT, deep vein thrombosis; POD, postoperative day. *Mixed-platform cohort; figures describe domestic platforms collectively.*

Table S4. Telesurgical (5G) network parameters and safety architecture

| **Study** | **Procedure (remote n)** | **Distance** | **Network parameters** | **Packet loss** |
| --- | --- | --- | --- | --- |
| Guo et al. [7] | Radical gastrectomy (20–27 remote) | 15 km | Total delay 226.2 ± 4.4 ms; round-trip 31.6 ± 3.8 ms | <0.1% |
| Chen et al. [6]† | Pancreaticoduodenectomy (1) | 900 km physical / 1035 km network | Mean delay 21 ms; total end-to-end delay <100 ms | NR |
| Yang et al. [10] | Cholecystectomy (20) | 70 km | Mean latency 43.4 ms (29.1–51.6); jitter 4 ms; upload 98.3 Mbps; download 213 Mbps | <1% |

*NR, not reported. † The remote pancreaticoduodenectomy was performed with the MP2000 platform, not the Toumai. Safety architecture (all remote programmes): standby bedside surgical team able to complete the procedure locally; deputy/second console; predefined conversion triggers; automatic switch of the robot to standby on signal loss. In Yang et al., a 3-second signal interruption triggered the master–slave standby mechanism without patient harm, and the operation was completed uneventfully.*

Table S5. Device-specification comparison: Toumai MT-1000 vs da Vinci Xi

| **Feature** | **Toumai MT-1000** | **da Vinci Xi** |
| --- | --- | --- |
| **Force-sensing function** | Yes (force-sensing trocar) | No |
| **Instrument diameter** | 8.4 mm | 8.5 mm |
| **Instrument uses (per instrument)** | 12–20 | 10–18 |
| **Vessel-sealing system** | No | Yes |
| **Robotic staplers** | No | Yes |
| **Immersive 3D resolution** | 1920 × 1080 | 1280 × 1024 |
| **Field of view** | 90° | 80° |
| **Picture-in-picture** | 2-input | 2-input |
| **ICG fluorescence imaging** | Yes | Yes |
| **Intelligent smoke evacuation** | Yes | No |
| **Monitor size** | 21.5 in | 19 in |
| **Operating force** | ≤0.1 N | ≤0.1 N |
| **Master–slave latency** | ≤50 ms | ≤80 ms |
| **Targeting accuracy** | 12° | 15° |
| **Dual surgeon console** | Yes | Yes |
| **Telesurgery support** | Multi-network (5G/4G/WAN/Wi-Fi) | No |
| **Simulation training system** | Yes | Yes |
| **Surgeon-console backup power** | 16 min | None |
| **Patient-cart backup power** | 16 min | 5 min |
| **Shoulder/boom actuation** | Manual drive | Motor drive |
| **Shoulder/boom joints (n)** | 4 | 5 |

*Reproduced and condensed from the device comparison reported by Tian et al. [9]. Every technical specification listed here is a manufacturer-declared value as compiled in that comparative device report [9]; none is derived from independent head-to-head clinical measurement. Comparative clinical outcomes, by contrast, are reported separately in Table S2 and are sourced from the primary comparative studies [9–11]. Specifications are manufacturer-declared and may vary by software version and instrument set; in particular, a declared force-sensing capability is not equivalent to clinically usable haptic feedback (see main Discussion).*

Table S6. ROBINS-I domain judgements with support for judgement

| **Study** | **Overall** | **Principal domains and rationale** |
| --- | --- | --- |
| Xu et al. [5] | Moderate | Single-arm retrospective design: uncontrolled confounding (D1) and consecutive-case selection (D2); unblinded outcome assessment (D6). No serious concerns for D3–D5/D7. |
| Chen et al. [6] | Serious | Intervention cannot be classified at the platform level (Toumai vs Edge MP) (D3); confounding from platform pooling (D1); platform-specific outcomes not reported. Other domains moderate. |
| Guo et al. [7] | Moderate | Prospective, registered single-arm trial with pre-specified endpoints; absence of a concurrent comparator (D1); unblinded outcomes (D6); small single-centre sample. |
| Zhang et al. [8] | Moderate | Exploratory single-arm design across mixed procedures; potential missing-data (D5) and outcome-measurement (D6) uncertainty; confounding (D1). |
| Tian et al. [9] | Moderate | Propensity-score matching reduced baseline imbalance, but residual confounding is possible (D1); retrospective, unblinded outcome assessment (D6). Low concern for D2–D5/D7. |
| Yang et al. [10] | Moderate | Prospective controlled trial with registration and pre-specified safety endpoints; small single-centre sample; unblinded outcomes (D6); confounding from non-randomised allocation (D1). |
| Wang et al. [11] | Moderate | Retrospective design with unequal groups (16 vs 34) raising selection concern (D2); unblinded outcomes (D6); small sample with possible missing data (D5). |

*ROBINS-I domains: D1 confounding; D2 selection of participants; D3 classification of interventions; D4 deviations from intended interventions; D5 missing data; D6 measurement of outcomes; D7 selection of the reported result. The case report [12] was not graded. Judgements reflect the review team’s independent assessment and should be verified at peer review.*
